# Phosphorylated TDP-43 (pTDP-43) aggregates in the axial skeletal muscle of patients with sporadic and familial amyotrophic lateral sclerosis

**DOI:** 10.1186/s40478-018-0528-y

**Published:** 2018-04-13

**Authors:** Matthew D. Cykowski, Suzanne Z. Powell, Joan W. Appel, Anithachristy S. Arumanayagam, Andreana L. Rivera, Stanley H. Appel

**Affiliations:** 1 Department of Pathology and Genomic Medicine, Houston Methodist Hospital, 6565 Fannin Street, Houston, TX 77030 USA; 2 Institute of Academic Medicine (IAM) in the Houston Methodist Research Institute (HMRI), Houston Methodist Hospital, 6565 Fannin Street, Houston, TX 77030 USA; 3 Houston Methodist Neurological Institute, Houston Methodist Hospital, 6565 Fannin Street, Houston, TX 77030 USA; 4 Department of Neurology, Houston Methodist Hospital, 6565 Fannin Street, Houston, TX 77030 USA

**Keywords:** Amyotrophic lateral sclerosis, Skeletal muscle, Inclusion body myositis, Paraspinous muscle, p62/ sequestosome-1, Autophagy, pTDP-43

## Abstract

Muscle atrophy with weakness is a core feature of amyotrophic lateral sclerosis (ALS) that has long been attributed to motor neuron loss alone. However, several studies in ALS patients, and more so in animal models, have challenged this assumption with the latter providing direct evidence that muscle can play an active role in the disease. Here, we examined the possible role of cell autonomous pathology in 148 skeletal muscle samples from 57 ALS patients, identifying phosphorylated TAR DNA-binding protein (pTDP-43) inclusions in the muscle fibers of 19 patients (33.3%) and 24 tissue samples (16.2% of specimens). A muscle group-specific difference was identified with pTDP-43 pathology being significantly more common in axial (paraspinous, diaphragm) than appendicular muscles (*P* = 0.0087). This pathology was not significantly associated with pertinent clinical, genetic (c9ALS) or nervous system pathologic data, suggesting it is not limited to any particular subgroup of ALS patients. Among 25 non-ALS muscle samples, pTDP-43 inclusions were seen only in the autophagy-related disorder inclusion body myositis (IBM) (*n* = 4), where they were more diffuse than in positive ALS samples (*P* = 0.007). As in IBM samples, pTDP-43 aggregates in ALS were p62/ sequestosome-1-positive, potentially indicating induction of autophagy. Phospho-TDP-43-positive ALS and IBM samples also showed significant up-regulation of *TARDBP* and *SQSTM1* expression. These findings implicate axial skeletal muscle as an additional site of pTDP-43 pathology in some ALS patients, including sporadic and familial cases, which is deserving of further investigation.

**Electronic supplementary material:**

The online version of this article (10.1186/s40478-018-0528-y) contains supplementary material, which is available to authorized users.

## Introduction

ALS is a progressive disorder characterized by motor neuron injury, muscle weakness, bulbar symptoms, such as dysarthria and dysphagia [1], and frontal-subcortical cognitive dysfunction in many patients [43, 44]. ALS has a lifetime risk of ~ 1 in 500 persons [51] and a prevalence in the United States reaching 17 per 100,000 individuals in the eighth decade [39]. Survival is generally poor and patients typically live only three to five years after diagnosis [49].

Pathologic studies of ALS nervous tissue have emphasized cell autonomous and non-cell autonomous pathologies in upper and lower motor neuron groups, and more recently, in non-motor brain regions [18, 38]. These include neuronal and glial inclusions comprising misfolded and phosphorylated TAR DNA-binding protein 43 kDA (pTDP-43) [10, 17, 35] and fused-in-sarcoma (FUS) protein [34], as well as dipeptide repeat pathology in C9ORF72 expansion-associated ALS (c9ALS) [54]. The critical role of glia and immune cells in disease progression is also recognized in human ALS and transgenic ALS models [26, 40, 58]. In contrast, since the original descriptions of ALS [16, 55], the secondary process of muscle atrophy following motor neuron loss and denervation has been emphasized. Indeed, electrophysiologic findings in ALS muscle are typically those of denervation atrophy [19, 29] and are accompanied by histopathologic changes of denervation, such as target fibers and fiber type grouping. At the same time, electrophysiological and functional studies of human ALS have supported an active and early role of skeletal muscle [48] and an early vulnerability of select muscle groups, such as paraspinous [29, 30]. Further, not all pathologies described in ALS muscle are those of denervation atrophy. Additional pathologies that have been described in some ALS muscle samples include myopathic features [28], fiber necrosis and inflammation [2], DNA fragmentation with expression of pro-apoptotic proteins [52], and reduced expression of mitochondrial proteins and mitochondrial DNA mutations/ deletions [4, 15, 56]. Studies of protein aggregates in human ALS muscle, that might indicate cell autonomous pathology, are much more limited. One study of 30 quadriceps biopsies did not identify pTDP-43 pathology in any samples [50]. Another recent study of deltoid samples found p62-positive, pTDP-43-negative inclusions [2] and p62-positive, pTDP-43-negative inclusions were recently described in the gastrocnemius of a single c9ALS patient [53].

Studies in ALS animal models have provided direct evidence that skeletal muscle can play a more active role in neurodegeneration. A variety of cell autonomous pathologies have been identified in the myofibers of transgenic (tg) mice expressing mutant Cu/Zn superoxide dismutase (mSOD1) [32]. These include increased endoplasmic reticulum stress response early in disease [12], reduction of heat shock protein expression [7], mitochondrial dysfunction [42], and pro-apoptotic signaling with increased cytosolic levels of calcium and calcium-binding proteins [13]. Aggregates of SOD1 protein have also been detected in the muscle of mSOD1 animals [6] and multiple studies have found an alteration in muscle expression of autophagy-related genes [14, 21]. The most compelling evidence to date for the active role of muscle in ALS is the finding that tg mice expressing G37R and G93A human SOD1 in skeletal muscle (hSOD1^mus^), but not in nervous tissue, develop neurologic and pathologic ALS [57], with the wildtype motor neurons developing ubiquitinated aggregates. Other muscle-specific tg models have also shown muscular atrophy and reduced strength [22] and motor neuron degeneration [23].

To further examine whether cell autonomous pathology is present in the muscle of human ALS patients, we studied 148 muscle specimens from 57 patients to identify the prevalence and muscle group distribution of pTDP-43, a well-characterized marker of ALS pathology in motor neurons. To extend the work of prior studies, we included samples not only of appendicular muscle (quadriceps, deltoid), but also diaphragm and paraspinous muscle groups. We also studied pTDP-43-positive samples to determine whether the autophagy-related protein p62/ Sequestosome-1 was present in myofiber inclusions. To determine the specificity of these findings, we compared the ALS samples to an additional 25 non-ALS muscle samples with neurogenic atrophy, including four samples of clinical and pathologic inclusion body myositis (IBM). Finally, we examined whether pTDP-43 pathology in muscle was associated with salient disease characteristics, including age, site of onset, disease duration, familial or c9ALS status, and the burden of nervous system TDP-43 pathology.

## Materials and methods

### ALS muscle samples and clinical data

The 57 ALS patients with tissue examined in this study all underwent autopsy at our institution. The sample was representative of clinically diagnosed ALS patients seen at our institution with (1) pathologic confirmation and (2) skeletal muscle samples obtained at autopsy from one or multiple muscle groups. All pathologically confirmed ALS cases with skeletal muscle available for study were included. The majority of patients were evaluated in the clinic of two study authors (SHA, JWA). The study was conducted with the approval of the Institutional Review Board at Houston Methodist Hospital (IRB-(3 N)-0114–0013).

Clinical variables recorded included (a) disease duration, defined as the time between first symptoms and autopsy date, (b) age at death, (c) site of disease onset (“limb”, “bulbar”, or “multifocal/other”), and (d) whether the patient had familial (fALS) or sporadic ALS (sALS). As previously described [17], c9ALS status was identified (or confirmed) for the purposes of this retrospective study by the presence of TDP-43-negative, p62-positive inclusions on immunofluorescence (purified mouse anti-p62 Ick ligand, 1:50, BD Biosciences, San Jose, CA) in hippocampus and/or cerebellar granule cells. All c9ALS cases were confirmed using poly-GA (MABN889, EMD Millipore, 1:200, mouse monoclonal) and poly-GP (ABN455, EMD Millipore, 1:1000, rabbit polyclonal) antibodies. In 42 of the 57 patients in this study, the percentage of nervous system regions-of-interest (ROIs) with TDP-43 pathology, as previously reported [17], was also available.

### Non-ALS muscle samples

Non-ALS patients (*n* = 25) included seventeen samples with neurogenic atrophy demonstrated on muscle biopsy. These non-ALS patients comprised four cases of biopsy-proven inclusion body myositis (IBM), one case with neurogenic atrophy in a patient with myasthenic symptoms, one case of denervation atrophy in a patient with nerve plexus injury, one case of steroid myopathy, and 10 biopsies with a range of mild neurogenic changes (scattered nuclear clumps, esterase-positive angulated and atrophic fibers). Eleven of the non-ALS biopsies were from quadriceps and the remainder were obtained from biceps (2), gastrocnemius (2), or an unspecified site (2). An additional eight samples were included that contained fairly abundant paraspinous muscle among the various tissues obtained at laminectomy and submitted for routine pathologic examination. Two such specimens were from cervical spine with the remainder from lumbar spine and diagnoses included synovial cyst (2), radiculopathy (1), spinal stenosis (4), and spondylolisthesis (1).

### Phospho(409/410)-TDP-43 immunohistochemistry

Phospho(409/410)-TDP43 antibody (1:500, rabbit polyclonal, 22309–1-AP, Proteintech, Rosemont, IL) was applied to all ALS samples, including paraspinous muscle (*n* = 44), deltoid (*n* = 34), diaphragm (*n* = 29), quadriceps (*n* = 26), skeletal muscle, NOS (*n* = 13), and one sample each of gastrocnemius and abdominal wall muscle. Immunostaining was performed on formalin-fixed, paraffin embedded (FFPE) tissue samples, sectioned at 4 μm, mounted on charged slides and dried for at least 2 h at 50–60 °C. Deparaffinization and rehydration were carried out using a series of xylenes, graded alcohols, and reagent grade water (ThermoFisher Scientific, Waltham, MA). Heat-based antigen retrieval was performed using a 1× antigen retrieval solution at pH 9 (Agilent Technologies, Santa Clara, CA) and was carried out for 1 h (30 min at 95 C^0^, followed by 30 min on ice). Subsequent washing steps were carried out using a commercial Tris-buffered saline solution (1×) containing Tween 20, pH 7.6 (Agilent Technologies) with a 3% hydrogen peroxide solution (VWR International, Radnor, PA) used to block endogenous peroxidase. Primary antibody was applied for a minimum of 1 h following a one-hour blocking step at room temperature with 2.5% horse serum (Vector Laboratories, Burlingame, CA). Slides were thoroughly washed and a species appropriate ImmPress™ HRP IgG detection kit (Vector Laboratories) was applied for 1 h at room temperature. Following additional washing steps, target antigen was visualized using DAB chromogen in substrate buffer (Agilent Technologies). Hematoxylin counterstain was applied and slides were taken to xylene and mounted with Permount™ (ThermoFisher Scientific). Negative and positive controls were used with all staining reactions. Positive controls comprised ALS spinal cord or medulla and performed appropriately.

### Evaluation of pTDP-43 inclusion pathology

Myofibers, adipose tissue, and neurovascular tissue were assessed for pTDP-43-reactive inclusions. For myofibers, only cases with unequivocal staining in the subsarcolemmal region or within sarcoplasm were recorded as positive (no intranuclear inclusions were identified). Semi-quantitation of pTDP43-positive samples was also performed. For pTDP-43 *density*, the number of involved fibers was quantitated in a single 100× low-power field (LPF). For pTDP-43 *extent*, the number of LPFs with pTDP-43 pathology per slide was expressed as a percentage of all LPFs with myofibers in the sample (e.g., 15 fields with pTDP-43 inclusion pathology of 25 total LPFs of muscle = 60% of fields with at least one pTDP-43 inclusion).

### p62 and FUS immunohistochemistry

Using the procedures described above, skeletal muscle samples that were positive for pTDP-43 were screened for inclusions of p62 (purified mouse anti-p62 Ick ligand, 1:100, BD Biosciences, San Jose, CA) and/or FUS/TLS (1:200, rabbit polyclonal, 11570–1-AP, Proteintech). Immunohistochemical procedures were identical to those described above. Negative and positive controls were used and performed appropriately. For p62, sections of spinal cord or medulla from ALS patients served as positive controls. For FUS, positive controls were sections of FTLD-FUS brain tissue.

### Additional staining in a subset of cases

In a subset of ALS cases with pTDP-43 inclusions, double labeling immunofluorescence procedures were performed to assess co-expression of pTDP-43 (as above) with fast myosin (anti-mouse, 1:400, Sigma Aldrich, M4276), slow myosin (anti-mouse, 1:1000, Sigma Aldrich, M8421), or p62 (as above). Samples were incubated overnight with a mixture of primary antibodies at 4 °C following deparaffinization and rehydration steps, antigen retrieval procedures (10 mM citrate buffer), and a blocking step using 2.5% horse serum. After several washes with phosphate buffered saline, secondary antibodies (1:200) were applied for 1 h, including the secondary antibodies Alexa Fluor® 555 anti-rabbit IgG (Thermo Fisher, A-21429), Alexa Fluor® 555 anti-mouse IgG (Thermo Fisher, A32727), Alexa Fluor® 488 anti-mouse IgG (Thermo Fisher, A11001), and Alexa Fluor® 488 anti-rabbit IgG (Thermo Fisher, A-11034). Following further PBS washes, sections were mounted using Vectashield Antifade mounting media with DAPI (H-1200, Vector Laboratories, Burlingame, CA).

Additional staining for N-terminal TDP-43 antibody (1:1000, rabbit polyclonal, 10782–2-AP, Proteintech, Rosemont, IL), which stains both physiologic and pathologic TDP-43, was performed in three ALS samples with pTDP-43 inclusions (see Results), two ALS samples without pTDP-43 inclusions, and a control sample of frontotemporal lobar degeneration with TDP-43 inclusion pathology. 1% Thioflavin S staining was performed in the same five ALS samples undergoing N-terminal TDP-43 immunohistochemistry, as well as four samples of IBM, three samples of non-ALS, non-IBM neurogenic atrophy, and two control samples (Alzheimer’s disease).

### Evaluation of immunofluorescence studies

All immunofluorescence studies were reviewed within 24 h of staining and images were captured in cellSens software 1.13 (Olympus America, Inc., Center Valley, PA) on an Olympus BX-43 Microscope using a DP71 camera, a EGFP FITC/Cy2 filter cube (set number 49002, Olympus, Center Valley, PA) and a CY3/TRITC filter cube (set number 49004, Olympus, Center Valley, PA). Slides were examined separately under DAPI, TRITC, and FITC filters, to facilitate examination of antibody/ fluorophore-specific signal versus structures with autofluorescence (e.g., pigment). Images were captured in cellSens software, including merged images combining all three channels.

### Electron microscopy of FFPE tissue

A protocol at our institution available for the ultrastructural study of specimens from FFPE blocks not fixed with glutaraldehyde was used to study four ALS muscle samples with comparatively extensive pTDP-43 inclusion pathology (see Results). A region of maximal pTDP-43 inclusion pathology was identified microscopically and marked on the tissue block face. A core of tissue was sampled from the corresponding tissue focus, deparaffinized and post fixed in buffered 2% osmium tetroxide. Tissues were dehydrated in a graded ethanol series, followed by resin infiltration with propylene oxide and EMbed 812 mixture (Electron Microscopy Sciences, Hatfield, PA). Samples were embedded overnight at 60 ^o^C. Semi-thick and ultrathin sections were collected using a LEICA EM UC7 (Leica Microsystems, Buffalo Grove, IL). Stained thin sections were imaged with JEOL 1400 transmission electron microscope (JEOL Ltd., Tokyo, Japan) and AMT Imaging Software (Advanced Microscopy Techniques, Corp., Woburn, MA).

### RNA isolation and real-time PCR

To examine *SQSTM1* (p62/ sequestosome-1) and *TARDBP* gene expression, RNA was isolated from four successive 10-μm sections of paraffin-embedded muscle tissue using an RNeasy® FFPE Kit (Qiagen, catalogue number 73504, Hilden, Germany) according to the manufacturer’s protocol. RNA was isolated from five ALS samples (p62 and pTDP-43-positive), three IBM samples (p62 and pTDP-43-positive), and three samples of non-ALS, non-IBM neurogenic atrophy (p62 and pTDP-43-negative). Quantification of RNA was performed using a Nanodrop ND 100 spectrophotometer (Thermo Fisher Scientific).

Real-time PCR primer assays were performed using SYBR® Green dye-based chemistry (Bio-Rad, catalogue # 1725151, Hercules, CA, USA) with commercially available and experimentally validated PCR primer pairs for *SQSTM1* (Bio-Rad, catalogue # qHsaCED0045925) and *TARDBP* (Bio-Rad, catalogue # qHsaCED0043888). Housekeeping genes utilized for normalization of transcript levels included *GAPDH* (Bio-Rad, catalogue # qHsaCED0038674) and *RPS18* (Bio-Rad, catalogue # qHsaCED0037454). Each real-time PCR experiment included eleven ALS, IBM, and atrophy muscle RNA samples plus a no template control (12 experimental wells per primer × 4 primers). This experiment was duplicated in the other half of the 96-well plate and then repeated, again in duplicate, using a new 96-well plate. A one-step real-time PCR protocol was performed on a CFX96 Touch (Bio-Rad, Hercules, CA, USA), including a melt curve step to exclude non-specific amplification. Experiments were analyzed in CFX Maestro™ software for Mac (Bio-Rad, Hercules, CA, USA). *TARDBP* and *SQSTM1* expression were normalized to both reference genes (*GAPDH*, *RS18*) and relative normalized gene expression was compared between patient groups. Significance testing was performed using an unpaired t-test.

### Descriptive statistics and statistical analyses

Fisher exact tests were used to determine the strength of association between pTDP-43 inclusion pathology in muscle fibers and individual muscle groups (deltoid, quadriceps, paraspinous, and diaphragm), as well as axial (diaphragm, paraspinous) versus appendicular (deltoid, quadriceps) musculature. Fisher exact tests were also used to examine the association between pTDP-43 inclusion pathology and c9ALS status, fALS status, and onset (limb versus bulbar). The strength of these associations was recorded by the *P*-value and odds ratio (OR).

Wilcoxon rank sum tests (Mann-Whitney U) were applied to examine the equality of medians in pTDP-43-positive and negative ALS patients with respect to age at death, disease duration, and percentage of central nervous system regions-of-interest (ROIs) positive for TDP-43 pathology [17]. As previously reported, the last of these measures was the percentage of 34 different brain regions positive for TDP-43 inclusions (using Proteintech 10782–2-AP) (ranging between 27.3 and 100%) and these data were available for 42 of the 57 patients studied here [17]. Similarly, Wilcoxon rank sum tests were applied to examine the equality of medians between IBM and ALS patients with respect to pTDP-43 density and pTDP-43 extent in positive skeletal muscle samples. Statistical testing was implemented in Matlab R2015b [37] and statistical significance was set at *P* ≤ 0.01.

## Results

### Demographics and clinical characteristics

ALS patient characteristics are listed in Table 1. Briefly, the 57 patients in this study comprised 39 men and 18 women with a median age of 61 years at death (interquartile range, IQR 56–68 years). Ten patients had a history of fALS (17.5%) and the remaining cases were sporadic (*n* = 45, 79%), or family history was unknown (*n* = 2). c9ALS was present in 13 patients (22.8%), accounting for 90% of the clinically designated fALS patients in this study and 4 sALS patients (8.9% of sALS cases). Limb onset was seen in 38 patients (66.7%), bulbar onset in 12 (21.1%), and onset was other/ multifocal in six (10.5%). Onset site was not available in one patient. Median disease duration was 2.99 years (1090 days, IQR 781–1565 days).

**Table 1:** Demographic, clinical, and sample characteristics in 57 ALS patients

Characteristic	Entire cohort ^a^
Age	61 years (IQR, 56–68), range 39–86 ^b^
Duration	1090 days (IQR, 781–1565), range 315–4886
Men/ Women	39 men (68.4%)/ 18 women (31.6%)
fALS ^c^/ sALS/ C9ALS	10 fALS (18.2%)/ 45 sALS (81.8%)/ 13 C9ALS
Onset site ^c^	38 Limb (66.7%)/ 12 Bulbar (21.1%)/ 6 Multifocal (10.5%)
Axial muscle samples	44 Paraspinous (29.7%)/ 29 Diaphragm (19.6%)
Append. Muscle samples	34 Deltoid (22.9%)/ 26 Quadriceps (17.6%)
Other muscle samples	13 NOS ^d^ (8.8%)/ 1 gastrocnemius/ 1 abdominus rectus

^a^Data presented are medians with interquartile range (IQR) in parenthesis or number with percentage

^b^For the 17 non-ALS samples with atrophy studied, median age was 60 years (IQR, 51–62). For the 8 non-ALS paraspinous samples, median age was 62 years

^c^fALS/ sALS and c9ALS status were not available in 2 patients. One patient had an unknown onset site

^d^These samples were obtained without reference to anatomic site and simply labeled as “muscle” in the original autopsy report

### Prevalence, distribution, and quantitation of pTDP-43 muscle pathology in ALS

**Fig. 1:**
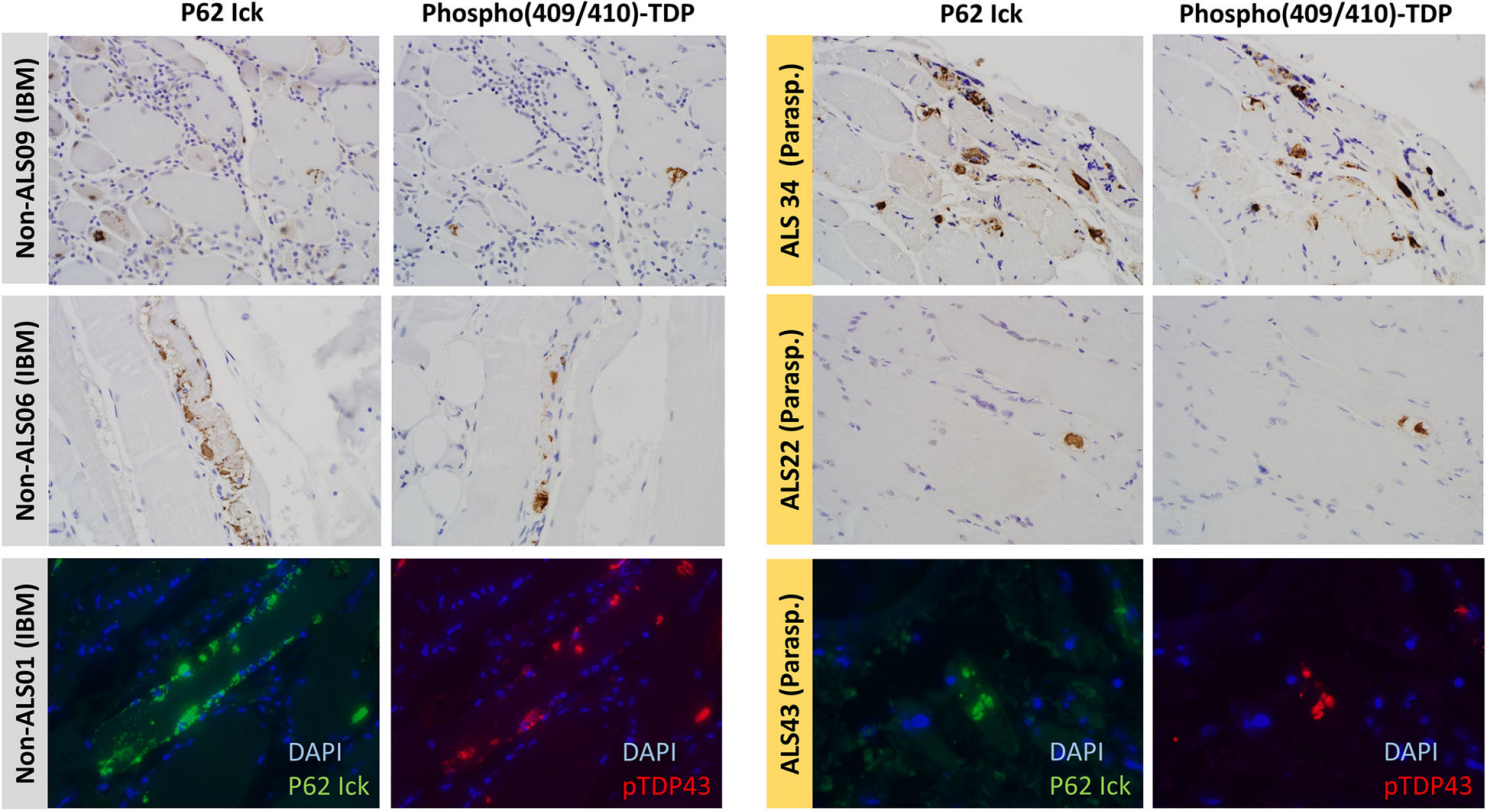
p62 Ick and pTDP-43 immunohistochemistry demonstrate p62-immunoreactive and pTDP-43-immunoreactive inclusions in three different IBM samples (left panels) and three different ALS samples (right panels; these examples all from paraspinous muscle). Immunofluorescence studies (bottom row) demonstrate co-localization of p62 Ick and pTDP-43 in both IBM and ALS samples, although p62 is the more sensitive of the two in detecting subsarcolemmal/ sarcolemmal inclusion pathology. Top two rows (immunohistochemistry) photographed at 400× and bottom row (immunofluorescence) photographed at 600×

**Fig. 2:**
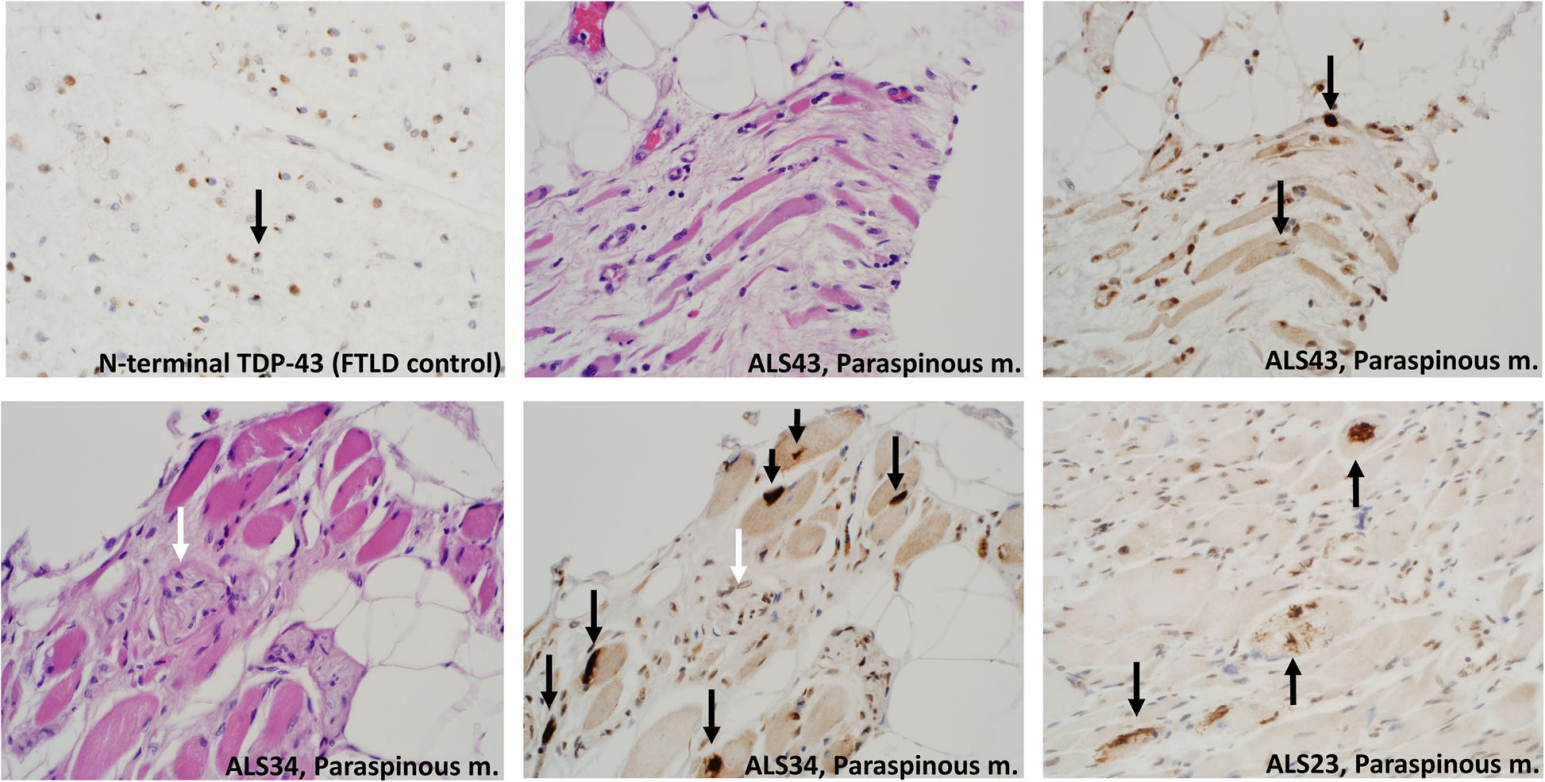
N-terminal TDP-43 immunohistochemistry in a control brain (frontotemporal lobar degeneration) and three ALS muscle samples shown to have pTDP-43-reactive inclusions. N-terminal TDP-43 immunohistochemistry reveals cytoplasmic inclusions (black arrows), as demonstrated separately with pTDP-43 immunohistochemistry. There is a loss of normal nuclear staining in affected myofibers. In sample ALS34 (bottom left) a small nerve is present (white arrow), which does not show pathologic staining in the adjacent panel (white arrow). All images are photographed at 400×

**Fig. 3:**
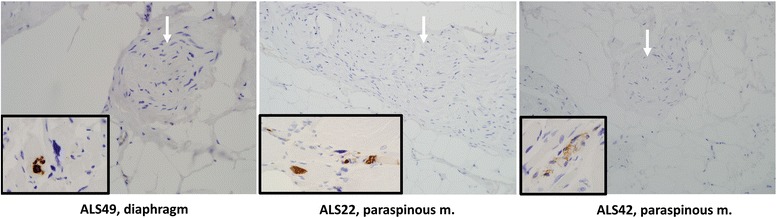
Three additional ALS samples (ALS49, ALS22, and ALS42) with pTDP-43 inclusion pathology in muscle fibers, but not in adjacent nerve that was readily found and evaluated in autopsy-derived ALS muscle specimens. Main panels of pTDP-43-negative nerve (white arrows) photographed at 200×. Inset of each panel, showing pTDP-43-positive myofibers in the same slide, photographed at 400×

pTDP-43 inclusion pathology was present in 24 of 148 ALS muscle specimens (16.2% of blocks) from 19 of 57 patients (33.3% of patients). pTDP-43 was identified in myofibers as cytoplasmic or subsarcolemmal aggregates, ranging from blocky, immunoreactive structures to dot- and dash-like and filamentous inclusions. Representative images of p62 and pTDP-43 pathology are shown in Fig. 1 for three IBM patients and three ALS patients. A subset of patients with and without pTDP-43 inclusions were studied and this showed N-terminal TDP-43 pathology in the same distribution as pTDP-43, with cytoplasmic staining and loss of normal nuclear staining (Fig. 2). Peripheral nerve elements were frequent components of the autopsy muscle specimens and no axonal or other neural pTDP-43 inclusions were identified in any samples, including those with pTDP-43 inclusions in muscle fibers **(**Fig. 3**)**. Intranuclear inclusions were not identified.

Quantitatively, a range of inclusion density and spatial extent was seen across pTDP-43-positive ALS samples. The median number of pTDP-43 inclusions per 100× field was 2 (IQR, 2 to 14.3 inclusions, range of 1 to 64). The majority of positive samples had multiple LPFs of pTDP-43 pathology, with a median of 5 LPFs per case having pTDP-43 inclusions (IQR, 2–7 LPFs, range of 1 to 15). A median of 10% of all microscopic LPFs that included skeletal muscle had pTDP-43 pathology (range of 3%–60%), reflecting that the inclusion pathology was patchy and not typically diffuse.

Co-localization of pTDP-43 inclusion pathology was examined with respect to fast- and slow-myosin heavy chain expression in 5 ALS cases and 1 IBM sample. In ALS cases, a median of 2 pTDP-43-immunoreactive inclusions was seen in the positive high-power field studied (range of 1–14). Phospho-TDP-43 inclusions in both slow- and fast-myosin positive fibers were seen in two cases (one with 78.5% in fast-myosin expressing fibers, the other with 80% in slow-myosin expressing fibers). The remaining ALS cases had pTDP-43 positive inclusions only in slow fibers (2 cases) or fast fibers (1 case). The single IBM sample tested had 83.3% of pTDP-43-positive inclusions in myofibers expressing fast myosin.

### p62 and FUS IHC in pTDP-43-positive ALS samples

All but one pTDP-43-positive ALS sample (23 of 24 samples) had pathologic inclusions of p62 in myofibers in the same distribution (Fig. 1). As in IBM cases, p62 was a more sensitive marker, revealing more extensive inclusion pathology than pTDP-43 alone. FUS immunohistochemistry was negative in all 24 ALS samples.

### Muscle group distribution of pTDP-43 positive samples in ALS

Among the 24 pTDP-43-positive ALS muscle samples, 10 were obtained from paraspinous musculature (41.7% of positive samples), 7 from diaphragm (29.2%), three from deltoid (12.5%), and one from gastrocnemius (4.2%). Three positive samples were from a muscle group not specified in the original autopsy report (12.5%) and were simply designated as “muscle, NOS.”

**Fig. 4:**
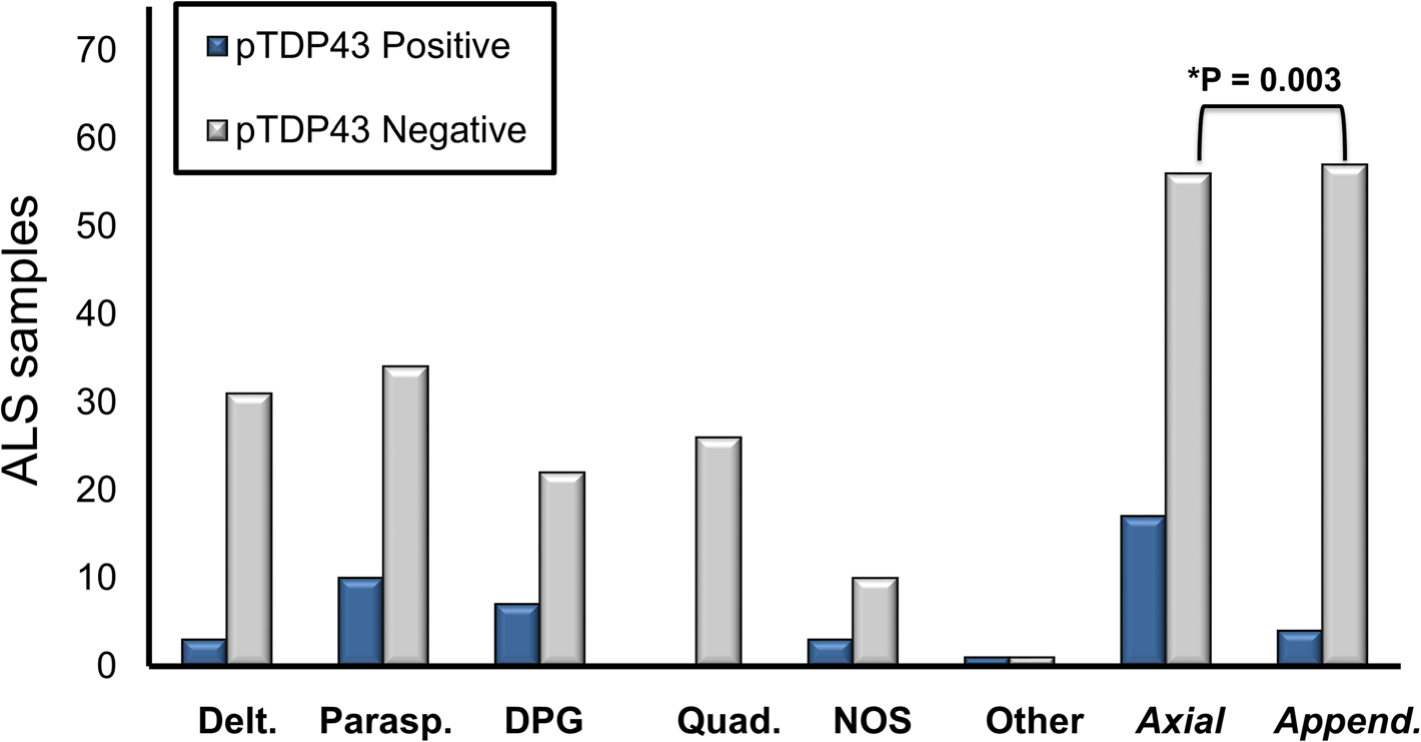
Distribution of pTDP-43 pathology in 148 samples from ALS patients, including (from left to right) deltoid, paraspinous, diaphragm, and quadriceps. “NOS” represents muscles that were not specified by muscle group in the autopsy report and “Other” includes one positive gastrocnemius sample and one negative abdominal wall sample. The right-most bars show that pTDP-43 pathology was significantly more prevalent in axial (diaphragm, paraspinous) than appendicular (deltoid, quadriceps) samples

Axial muscle groups represented 17 of 24 positive samples (70.8%) (Fig. 4). Fisher’s exact test revealed a correspondingly strong and significant positive association between pTDP-43 pathology in ALS patients and axial musculature (paraspinous, diaphragm) versus appendicular muscle groups (*P* = 0.0092, OR = 4.25). No significant pTDP-43 and individual muscle group association (positive or negative) was seen for deltoid (*P* = 0.2279, OR = 0.44), quadriceps (*P* = 0.07, OR = 0.17), or for paraspinous (*P* = 0.14, OR = 2.1) or diaphragm (*P* = 0.25, OR = 2.04) considered separately (Fig. 4).

Among the 19 ALS patients with any pTDP-43-positive muscle sample, 4 patients had multiple pTDP-43-positive samples (7% of the cohort, 21% of positive samples). The combinations of pTDP-43-positive samples included: diaphragm and paraspinous (2 patients), diaphragm, paraspinous, and deltoid (1 patient), and diaphragm and deltoid (1 patient). Three of these patients had clinically-designated sALS (75%) and c9ALS was present in two of these four (50%).

### Clinical and pathologic associations of pTDP-43 muscle pathology

The characteristics of ALS patients with and without pTDP-43-positive muscle samples are shown in Table 2. Briefly, patients with pTDP-43 skeletal muscle pathology (*n* = 19; 13 males, 6 females) had a median age of 64 years (IQR, 58.5–69.5 years), median disease duration of 1114 days (IQR, 840 to 2133.5 days), and included three fALS and four c9ALS patients. Patients had limb (*n* = 11), bulbar (5), and multifocal (3) sites of symptom onset. The group without pTDP-43 pathology had a median age of 59.5 years (IQR, 54.3–66.5 years), median disease duration of 1085 days (IQR, 723 to 1390 days), and included 7 fALS and 9 c9ALS patients. These pTDP-43-negative patients had limb (*n* = 27), bulbar (7), and multifocal (3) sites of symptom onset.

**Table 2:** Characteristics of muscle samples with and without pTDP-43 pathology

Variable	pTDP-43 Positive (*n* = 19)	pTDP-43 Negative (*n* = 38)	*P-*val ^a^
Muscle group	17 Axial, 4 Appendicular ^b^	56 Axial, 57 Appendicular ^b^	*0.003*
Patient age	64 years (IQR, 58.5–69.5)	59.5 years (IQR, 54.3–66.5)	0.1
Disease duration	1114 days (IQR, 840–2134)	1085 days (IQR, 723–1390)	0.34
CNS TDP^+^ ROI ^c^	58.3% TDP+ (IQR, 45.5–79.4)	50.0% TDP+ (IQR, 42.3–76.5)	0.67
Men, Women	13 men, 6 women	26 men, 12 women	*NA*
fALS, sALS	3 fALS, 16 sALS	7 fALS, 29 sALS	0.78
C9ALS ^d^	4 C9ALS	9 C9ALS	0.80
Onset site	11 Limb, 5 Bulb, 3 Multifocal	27 Limb, 7 Bulb, 3 Multifocal	0.49

^a^*P*-values represent differences using Fisher exact test or Mann-Whitney U test as appropriate (See Methods/ Results). ^b^ These numbers do not include three positive “NOS” samples, ten negative “NOS” samples, or negative abdominal wall muscle. ^c^ The percentage of brain and spinal cord ROIs positive for TDP-43 pathology as previously described in Cykowski et al., 2017 [17] (see Methods). ^d^ C9ALS status accounted for 9 of 10 fALS patients with one patient having an unknown genetic basis

No significant differences were identified between ALS patients with and without pTDP-43 muscle pathology with respect to age (z = 1.7, *P* = 0.1) or disease duration (z = 0.95, *P* = 0.34). Likewise, no significant association was seen between pTDP-43 status in muscle and fALS status (OR = 0.78), c9ALS status (OR = 0.8), or site of symptom onset (OR = 0.57). Utilizing data from an earlier study [17], ALS cases with pTDP-43 muscle pathology had a median of 58.3% and 50.0% of brain regions involved by TDP-43 inclusion pathology, respectively (z = 0.42, *P* = 0.67).

### Inclusion pathology in IBM and non-IBM, non-ALS muscle

**Fig. 5:**
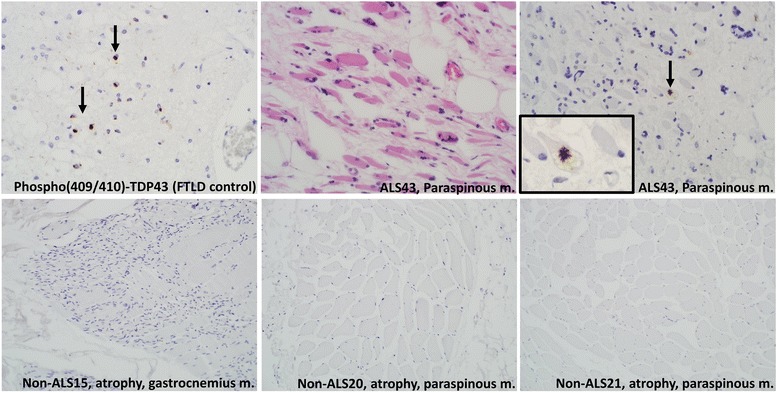
Non-ALS, non-IBM samples were negative for pTDP-43 pathology. Representative brain section control from a patient with frontotemporal lobar degeneration (top left panel), stained for pTDP-43, followed by H&E-stained section and positive pTDP-43 myofiber in an ALS patient. Bottom panels show pTDP-43 negative results in a patient with long-term steroid use and profound fiber atrophy and two samples of pTDP-43-negative paraspinous muscle obtained in the course of laminectomy in non-ALS patients. Images in the top row all photographed at 400×, except for the inset of pTDP-43 staining at the top right (600×) for sample ALS43. Images in the bottom row all photographed at 200×

Non-ALS muscle biopsies with atrophy (*n* = 17) included 9 females and 8 males with a median age of 60 years (IQR, 51–62 years). Samples of non-ALS paraspinous muscle (*n* = 8) included 6 females and 2 males with a median age of 62 years (IQR, 58–66 years). Among non-ALS samples, only IBM cases showed pTDP-43-immunoreactive inclusions (see Figs. 1 and 5).

Four cases of IBM (clinically suspected and pathologically confirmed) were from three males and one female with a median age of 59 years. Among these samples, only IBM cases (100%) had pTDP-43 inclusions, which were also p62-immunoreactive (Fig. 1). Three non-ALS, non-IBM cases with mild neurogenic atrophy had granular, non-specific p62 immunoreactivity (pTDP-43 negative) that did not resemble that seen in either ALS or IBM patients. Likewise, in non-ALS paraspinous samples, p62 occasionally showed immunoreactive structures, but these were uniformly pTDP-43-negative and did not resemble those in ALS or IBM muscle. In IBM patients, within the focus of maximum pTDP-43 pathology (100× field), IBM cases had a median of 10 involved myofibers (IQR of 7–11.8), whereas for ALS cases the median was 2 (IQR of 2–14.3) (z = − 1.1, *P* = 0.26). However, IBM and ALS patients with pTDP-43 inclusions did significantly differ in the spatial extent of pathology, with IBM patients having a median of 67% of LPFs involved (z = − 2.7, *P* = 0.007).

### pTDP-43 replication study in a subset of cases

A subset of 5 pTDP-43-positive muscle samples, two from patients with IBM and three from patients with ALS, were sent to an independent research laboratory for separate evaluation using their pTDP-43 immunohistochemistry protocol (phospho-TDP-43, 1D3 clone, 1:500). Five unstained sections, cut at 4 μm and labeled “ALS1–3” and “IBM1–2”, were shipped to the laboratory. Their results matched the spatial distribution and staining intensity of pTDP-43 in all IBM and ALS samples (see Online Resource Additional file 1: Figure S1).

### Ultrastructural findings

**Fig. 6:**
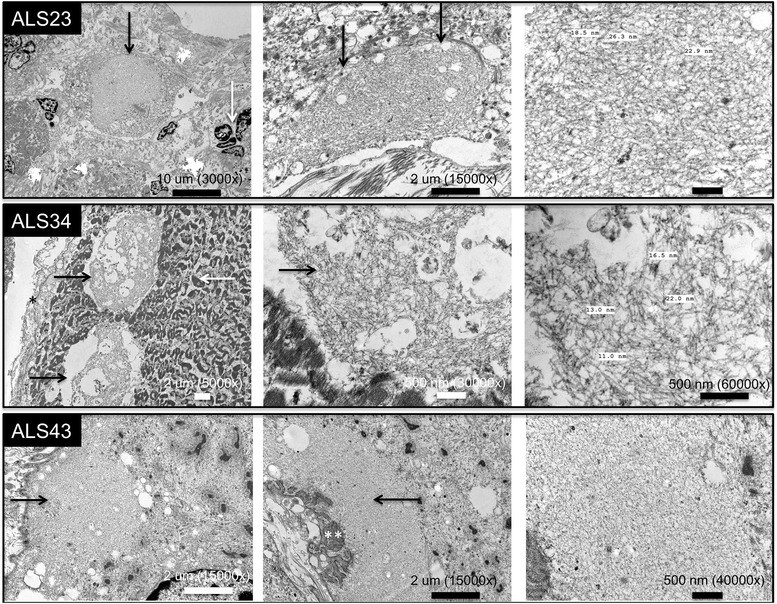
Electron microscopy of three ALS muscle samples (study samples ALS23, ALS34, and ALS43). For each muscle sample, a region-of-interest was dissected out of the FFPE block in a region with maximal pTDP-43 and p62 inclusion pathology and subsequently processed for electron microscopy using a protocol for FFPE specimens (see Methods). Filamentous material (black arrows in all three rows) is present in these foci, entirely within myofibers and sharply demarcated from degenerating myofibrils (white arrow, middle row, left-most panel). Basement membrane (black asterisk, middle row, left panel) and nuclear clumps (white arrow, top row, left panel) are also identified, despite the degenerated nature of the specimen. The filamentous material identified predominantly measures between 10 and 20 nm in thickness (right panels of top and middle rows, which are enlargements of the middle panels in their respective rows). In the bottom row, the middle and right-most panels show accumulation of this material adjacent to invaginations of the cell membrane at an apparent neuromuscular junction (white asterisks). Scale bar and magnifications are shown for each panel

A region of FFPE tissue, enriched for pTDP-43 pathology in ALS muscle samples, was prepared for electron microscopy (see Methods). Due to the nature of the specimen, autolytic changes and degradation of the sarcomere were present, as evidence by degeneration of myofibrils and Z-bands (Fig. 6). However, in each sample studied, sarcolemmal or subsarcolemmal filamentous material, ranging between 10 and 20 nm in width, was identified. This material was well demarcated from degenerating sarcomeres and is not identified in specimens with non-specific neurogenic atrophy or autolysis (personal experience of the authors). The structures resembled non-specific filamentous material that may be seen in muscle biopsy (filamentous bodies), although this material was more frequent in pTDP-43-positive ALS samples submitted for electron microscopy than we have observed for filamentous bodies in clinical muscle biopsy samples. Figure 6 shows the appearance of this material in sarcolemmal (top row), subsarcolemmal (middle row) and subsarcolemmal locations adjacent to a neuromuscular junction (bottom row), in three different patient samples. In this limited sample, no intranuclear inclusions, myelin-like figures, or lysosomal debris was identified. Mitochondria were too degenerated for evaluation.

### Quantitative PCR

**Fig. 7:**
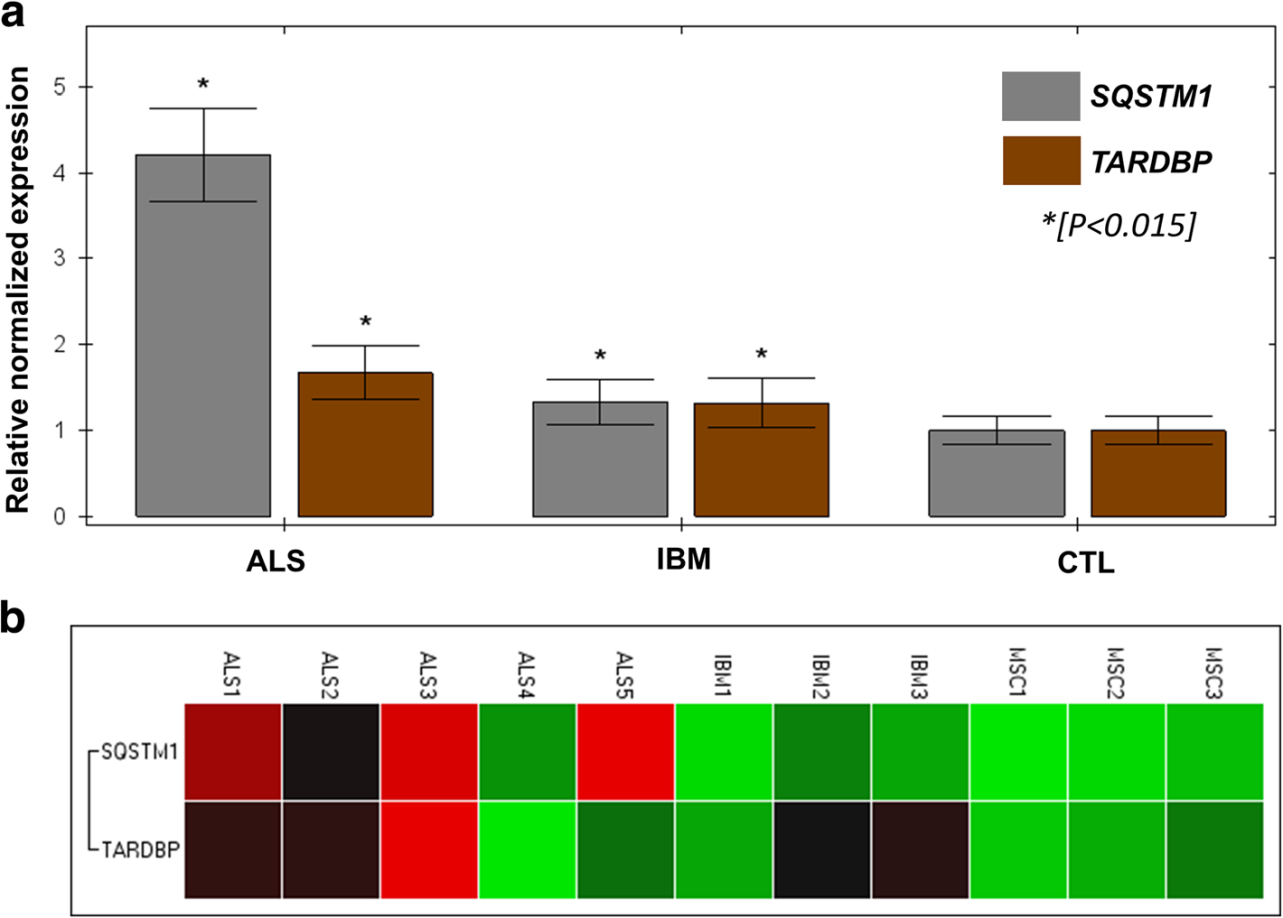
qPCR analysis was performed in 5 ALS muscle samples (“ALS1–5”) and 3 IBM muscle samples (“IBM1–3”), all containing p62 and pTDP-43 inclusions by immunohistochemical studies (see Results). Three non-ALS, non-IBM samples with mild neurogenic atrophy in the muscle biopsy were also studied (“MSC1–3”). *SQSTM1* and *TARDBP* were analyzed relative to the expression of two housekeeping genes (*18 s*, *GAPDH*) and data shown are combined from two 96-well plates (4 replicates per sample and primer). There was a significant increase in relative normalized gene expression for both ALS and IBM samples (**a**), relative to controls (right) and this was most significant for *SQSTM1* expression in ALS samples (> 4-fold expression relative to controls, *P* < 0.0001). Clustergram by gene target (**b**) shows heterogeneity across ALS and IBM samples with increase gene expression (red-brown) most conspicuous in ALS samples 1–3 and 5, as well as IBM samples 2 and 3

As shown in Fig. 7**,** real time PCR analyses revealed increased *TARDBP* and *SQSTM1* mRNA levels in both ALS and IBM patients relative to non-ALS, non-IBM control muscle. For ALS samples, relative to controls, there was a 4.21 fold increase (expression standard error of the mean (SEM) of 0.54) (P = < 0.0001) in *SQSTM1* and 1.67 fold increase in *TARDBP* (SEM = 0.31) (*P* = 0.0128). For IBM samples, relative to controls, there was a 1.33 fold increase in *SQSTM1* expression (SEM of 0.26) (P = 0.012) and 1.31 fold increase in *TARDBP* expression (SEM of 0.29) (*P* = 0.014).

## Discussion

This study demonstrates that aggregates of phosphorylated TDP-43 may be identified in the skeletal muscle of both sALS and fALS patients, including patients with and without c9ALS. This implicates axial skeletal muscle as an additional site of pTDP-43 pathology in ALS. A muscle group-specific difference in muscle pathology was also suggested by the finding that pTDP-43 inclusions were significantly more frequent in samples from axial muscle groups than appendicular groups (the absence of inclusion pathology in quadriceps samples is also consistent with the negative result of an earlier study [50] that did not assess axial muscle groups). Our finding that pTDP-43-positive (FUS-negative) aggregates in ALS samples are also positive for the autophagy pathway protein p62/ sequestosome-1 suggests the possibility of an engagement of endogenous autophagic mechanisms in ALS muscle, as in motor neurons. Indeed, this pattern resembled the co-localization of pTDP-43 and p62 inclusions in IBM [9, 31], an intrinsic myopathy in the differential diagnosis of ALS [33] with pathologic protein aggregates, progressive and asymmetrical weakness [46], and impairments in autophagy. Other characteristic features of IBM, however, were not identified in our ALS samples and there was significantly more extensive pTDP-43 inclusion pathology in IBM muscle than in ALS. Nonetheless, pTDP-43 aggregates in ALS and IBM muscle may arise through similar mechanisms, including impairments in autophagy and proteostasis.

To our knowledge this is the first study to systematically demonstrate the presence of pTDP-43 aggregates in the myofibers of ALS patients, particularly in axial muscle groups. This implicates ALS muscle as an additional site of pTDP-43 pathology, as previously demonstrated in motor neurons, non-motor neurons, and glia. The downstream effects of cytoplasmic pTDP-43 pathology in ALS muscle cells requires further study, though studies in transgenic animals expressing mutant human TDP-43 have identified a toxic gain-of-function from cytoplasmic aggregation, leading to transcriptional dysregulation, including within histone processing genes [3]. Several results of this study raise the question as to whether pTDP-43 in ALS muscle represents a cell autonomous pathology. First, the presence of pTDP-43 inclusions in muscle did not associate at all with disease duration or the burden of central nervous system TDP-43 pathology [17]. If cell-to-cell spread were the sole mechanisms explaining muscle pTDP-43 pathology, one would expect significant positive associations between muscle fiber inclusion pathology and either duration or the burden of nervous system TDP-43 pathology. Second, pTDP-43 co-localized with p62 in affected muscle fibers and a concomitant up-regulation of *TARDBP* and *SQSTM1* gene expression was seen by real-time PCR. Co-localization with an autophagy pathway protein (p62), and up-regulation in gene expression (for TDP-43 and p62), suggests this may be an endogenous pathology in muscle that engages the autophagy pathway. Increased expression of p62 has also been seen in non-ALS, non-IBM muscle diseases, including genetically driven forms of muscular dystrophy [20], so it may not implicate autophagy in all cases. Third, pTDP-43 co-localized with both fast- and slow-myosin expressing fibers in the same ALS sample (motor neurons innervating fibers of a single type) and was distributed within multiple fascicles rather than a single fascicle. However, it is also important to recognize that the majority of skeletal muscle samples were negative for pTDP-43. One reason for this may be that our study used single tissue samples obtained from between one and four muscle groups per patient. This approach is distinctly different from the postmortem examination of ALS nervous systems, where all anatomic regions (brain, spinal cord) are amenable to sampling and diagnostic pTDP-43 that may actually be quite limited in spatial extent. Recent autopsies at our institution, not included in this study, with paraspinous samples at multiple spinal levels, have indeed shown patchy, multifocal and segmental involvement of muscle (see Online Resource Additional file 1: Figure S2). This suggests that the true prevalence of pTDP-43 pathology in muscle cannot be ascertained from single biopsy-size samples and may be underestimated even in the present study.

An alternative mechanism explaining pTDP-43 aggregates in ALS muscle is a “prion-like” transfer of pTDP-43 protein, possibly through anterograde axonal transport. This model of neuron-to-neuron spread has been proposed to explain dissemination of pTDP-43 pathology in brain and spinal cord [8]. If trans-synaptic spread between neurons indeed takes place in ALS, then spread along the axons of motor neurons, across the neuromuscular junction, and into myofibers is plausible. A distance-dependent interaction between motor neurons and muscle, possibly acting in concert with muscle-specific pathology, may explain the enrichment of pTDP-43 inclusions in axial muscle. This question deserves further investigation, possibly through studies examining the density of spinal cord motor neuron pTDP-43 pathology in tandem with paraspinous muscle samples at the same spinal level.

Our study also identified co-localization of p62/ sequestosome-1 in pTDP-43 inclusions of ALS muscle, which suggests the possibility that the autophagy pathway is activated. This recapitulates pathology well described in inclusion body myositis (IBM). Further comparison of ALS and IBM muscle samples may provide insight as to how pTDP-43 inclusions arise in vulnerable ALS muscle groups. To date, there have been few studies of pTDP-43 (or p62) pathology in ALS muscle. None of these, to our knowledge, have examined both axial and appendicular muscle groups or compared findings in ALS and IBM, and non-ALS, non-IBM samples with neurogenic atrophy. Previous studies do include a negative study of ALS quadriceps biopsies from 30 patients [50] (the 26 quadriceps samples studied here were also negative), a study of 31 deltoid biopsies finding p62-immunoreactive, pTDP-43-negative foci in 25.8% of samples [2], and a case report of a c9ALS patient with blocky p62 inclusions in gastrocnemius [53]. Inclusion pathologies have been much more extensively studied in IBM, where β-amyloid, α-synuclein, and p-tau inclusions have also been described [5]. IBM inclusions are also reported to be positive for Thioflavin S, a finding identified here in all four IBM samples studied and in three pTDP-43-positive ALS samples examined (see Online Resource Additional file 1: Figure S3). In IBM, p62 appears to be the most sensitive marker of inclusion pathology [27] and these additional inclusions have not been seen in all studies [9]. Nonetheless, these findings have led to the hypothesis that protein aggregates in IBM muscle result from impairments in autophagy, the cellular process by which misfolded protein aggregates are degraded [25, 31]. Supporting this hypothesis are the increased expression of the endosome marker clathrin, the autophagy-related protein ATG5, the microtubule-associated protein light chain (LC3), and beclin-1 [25] in IBM samples. IBM samples also demonstrate more frequent LC3-reactive fibers than in pTDP-43-negative, non-IBM inflammatory myopathy (e.g., polymyositis), which indicates that alterations in autophagy are critical in IBM and not linked to inflammation per se. For ALS, autophagy impairments in motor neurons, as in IBM muscle fibers, have been proposed as a mechanism leading to cell degeneration and toxic protein aggregates [11, 45]. Moreover, mutations in fALS have been reported in autophagy-related genes, including optineurin (*OPTN*) [36], ubiquilin 2 (*UBQLN2*), sequestosome 1 (*SQSTM1*) [24], and ubiquitin segregase (*VCP*) [51] (the last of these also being described in familial IBM). In ALS nervous systems, p62- and LC3-reactive inclusions have also been identified in spinal cord motor neurons [47], in addition to TDP-43 [41]. The findings of the current study, which extends the spectrum of pTDP-43 and p62 inclusion pathology to ALS skeletal muscle, particularly in axial muscles, suggests that impairments in autophagy may also involve ALS muscle. Future studies are needed to determine whether other autophagy-related proteins, such as LC3, beclin-1, and optineurin, are over- or under-expressed in ALS muscle.

An intriguing finding of this study was that pTDP-43 pathology was significantly enriched in axial muscle samples. It is known that physiologic abnormalities in paraspinous muscle by electromyography (EMG) are reliable in confirming the clinical diagnosis of ALS and help to distinguish the disease from its mimics [29]. Paraspinous abnormalities have also been described as being present early in the disease [30]. Denervation changes by EMG in paraspinous muscle are also associated with features of diaphragm denervation – an association possibly explained by the medial position of the relevant motor neurons in the ventral horn [19]. A recent study of multiple muscle groups, including thoracic (T10), lumbar (L3, L5), and sacral (S1) paraspinous muscle has also shown non-contiguous abnormalities by needle EMG (e.g., fibrillation potentials). This finding suggests the possibility of multifocal and discontinuous initiation of disease in different motor neuron pools. More extensive sampling of paraspinous muscle groups at autopsy will help to determine if pTDP-43 pathology is similarly multifocal and discontinuous. Further, a limitation of this study, to be addressed in future studies, is that autopsy-derived diaphragm and paraspinous muscle samples from non-ALS patients with other critical illnesses or neurological diseases were not available for study. Paraspinous muscle and muscle biopsy specimens from a range of non-ALS, non-IBM neurologic diagnoses did not show pTDP-43 pathology, but these specimens were not from patients with critical illnesses, such as ALS.

Studies in the mSOD1 mouse model of ALS have provided direct evidence that cell autonomous pathology in muscle can drive the development of the disease [32]. The muscle of mSOD1 animals has been reported to have alterations in the endoplasmic reticulum stress response [12] and heat shock protein expression [7], as well as mitochondrial dysfunction [42], pro-apoptotic signaling, and increased cytosolic levels of calcium and calcium-binding proteins [13]. Aggregates of SOD1 protein have also been detected in gastrocnemius, soleus, and flexor digitorum longus of the transgenic animal [6]. Further, as in IBM, increased gene expression of autophagy components LC3 and SQSTM1 is reported in mSOD1 muscle [14]. Most critically, studies of transgenic ALS mice have revealed that muscle-restricted and muscle-initiated pathologies may induce motor neuron degeneration. Specifically, expression of G37R and G93A human SOD1 in skeletal muscle (hSOD1^mus^), but not nervous tissue, can lead to neurologic and pathologic ALS [57] with ubiquitinated aggregates in wild type neurons. Models with a muscle-restricted distribution of *SOD1* G93A mutation also show progressive muscular atrophy, mitochondrial dysfunction, and reduced muscle strength [22] and the atrophic muscles of these mice up-regulate autophagy genes, including *LC3*.

## Conclusions

In summary, this study implicates axial skeletal muscle (diaphragm and paraspinous muscles) as an additional site of pTDP-43 pathology in ALS. Up-regulation of *TARDBP* and *SQSTM1* expression by real-time PCR supports this as a possible cell autonomous pathology in ALS, though future studies using unbiased techniques with a wide range of transcripts for normalization are needed (e.g., RNA-Seq). Additional studies utilizing tissue dedicated for electron microscopy study, with registration to histologic data, are required to clarify the ultrastructural features of the pTDP-43-positive material in ALS muscle. Nonetheless, our findings suggest that the impaired clearance of misfolded proteins in ALS, a well-recognized mechanism in motor neuron degeneration, may play an important role in pTDP-43 pathology in ALS muscle.

## Additional file

Additional file 1: **Figure S1.** ALS muscle samples from three patients (top, middle, and bottom rows) with pTDP-43-immunoreactive inclusions. pTDP-43-stained sections (Proteintech clone 22309-1-AP) and H&E-stained sections from Houston Methodist (lab of MDC) are shown in the left three panels for each patient. The right-most panel of each patient shows repeat pTDP-43 staining at the University of Kentucky using the protocol of that laboratory and a different pTDP-43 antibody (1D3 clone, 1:500). All images are photographed at 400x without magnification except for five images photographed at 200x (top row, two right-most panels; middle row, H&E section; bottom row, two left-most panels). **Figure S2.** Four muscle samples from a recent autopsy of an ALS patient not included in this study. The patient had a clinical diagnosis of C9ALS and a positive family history of ALS (fALS). Examination of brain and spinal cord confirmed ALS and showed characteristic p62-immunoreactive and TDP-43-negative lesions in brain, consistent with C9ALS. Examination of seven muscle samples using p62 and pTDP-43 immunohistochemistry showed unequivocal pTDP-43-reactive lesions in muscle fibers of thoracic paraspinous and diaphragm muscle, an equivocal focus in sacral paraspinous muscle (not shown), but no pTDP-43-positive lesions in cervical paraspinous muscle (not shown). The inset of diaphragm shows the same focus on a deeper histologic section, also stained for pTDP-43, further revealing the thread- and dash-like inclusions in this sample. All images are photographed are 200x except for diaphragm, which is photographed at 400x. **Figure S3.** 1% Thioflavin S staining was performed in Alzheimer’s disease (AD) control tissue from occipital cortex and hippocampus/ subiculum (top left panel, 200x, and top middle panel, 400x), IBM muscle (top right, Non-ALS09, photographed at 600x), pTDP-43-positive ALS muscle samples with electron microscopy data shown in Fig 6 (ALS43, ALS34, ALS23), all photographed at 600x magnification, and a pTDP-43-negative ALS muscle with atrophy and abundant lipofuscin pigment (ALS50) (600x). Green arrows highlight thioflavin S-reactive material in all samples, whereas white arrows indicate autofluorescent material, such as lipofuscin pigment (top left, bottom left, and bottom right panels), which appears yellow-orange in images combining FITC/ DAPI/ and TRITC filters (see Methods for detail on immunofluorescence microscopy). For ALS sample ALS43, the inset at lower left (600x) shows the similar shape of inclusions in this area by pTDP-43 immunohistochemistry. (PDF 1807 kb)
