# Supplementary material for: Phosphorylated TDP-43 (pTDP-43) aggregates in the axial skeletal muscle of patients with sporadic and familial amyotrophic lateral sclerosis
Source: Acta Neuropathol Commun. 2018 Apr 13;6:28. doi: 10.1186/s40478-018-0528-y (PMC5899326; doi:10.1186/s40478-018-0528-y)
Supplement: Supplementary file 1 — Figure S1. ALS muscle samples from three patients (top, middle, and bottom rows) with pTDP-43-immunoreactive inclusions. pTDP-43-stained sections (Proteintech clone 22309-1-AP) and H&E-stained sections from Houston Methodist (lab of MDC) are shown in the left three panels for each patient. The right-most panel of each patient shows repeat pTDP-43 staining at the University of Kentucky using the protocol of that laboratory and a different pTDP-43 antibody (1D3 clone, 1:500). All images are photographed at 400x without magnification except for five images photographed at 200x (top row, two right-most panels; middle row, H&E section; bottom row, two left-most panels). Figure S2. Four muscle samples from a recent autopsy of an ALS patient not included in this study. The patient had a clinical diagnosis of C9ALS and a positive family history of ALS (fALS). Examination of brain and spinal cord confirmed ALS and showed characteristic p62-immunoreactive and TDP-43-negative lesions in brain, consistent with C9ALS. Examination of seven muscle samples using p62 and pTDP-43 immunohistochemistry showed unequivocal pTDP-43-reactive lesions in muscle fibers of thoracic paraspinous and diaphragm muscle, an equivocal focus in sacral paraspinous muscle (not shown), but no pTDP-43-positive lesions in cervical paraspinous muscle (not shown). The inset of diaphragm shows the same focus on a deeper histologic section, also stained for pTDP-43, further revealing the thread- and dash-like inclusions in this sample. All images are photographed are 200x except for diaphragm, which is photographed at 400x. Figure S3. 1% Thioflavin S staining was performed in Alzheimer’s disease (AD) control tissue from occipital cortex and hippocampus/ subiculum (top left panel, 200x, and top middle panel, 400x), IBM muscle (top right, Non-ALS09, photographed at 600x), pTDP-43-positive ALS muscle samples with electron microscopy data shown in Fig 6 (ALS43, ALS34, ALS23), all photographed at 600x magnification [file 40478_2018_528_MOESM1_ESM.pdf]

Online Resource Additional file 1

Article: “Phosphorylated TDP-43 (pTDP-43) aggregates in the axial skeletal muscle of patients with sporadic and familial amyotrophic lateral sclerosis”

Journal: *Acta Neuropathologica Communications*

Authors: Matthew D. Cykowski, MD, Suzanne Z. Powell, MD, Joan W. Appel, PA-C, Anithachristy S. Arumanayagam, Andreana L. Rivera, MD, and Stanley H. Appel, MD

Corresponding author: Matthew D. Cykowski, M.D., Houston Methodist Hospital, 6565 Fannin Street, Houston, TX, 77030. Email: mdcykowski@houstonmethodist.org.

This PDF file includes:

Additional file 1: **Figures S1-S3**

Online Resource Additional file 1

Figure S1. Repeat staining of pTDP-43-positive ALS samples.

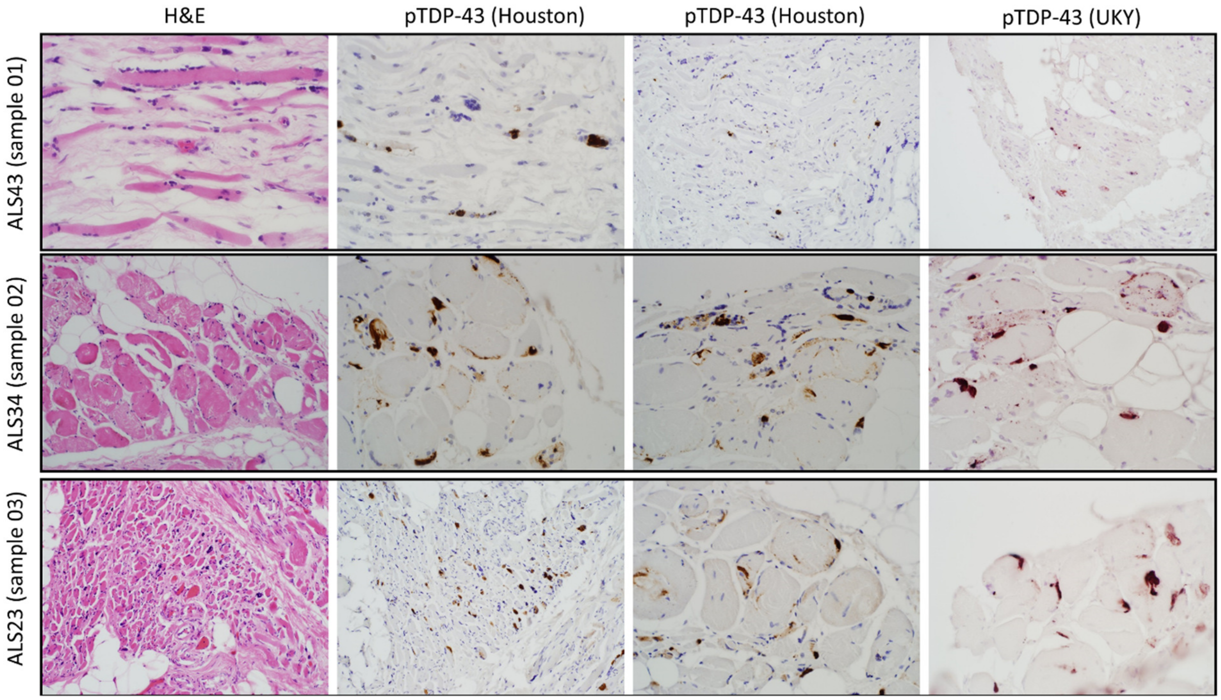

Figure S1 ALS muscle samples from three patients (top, middle, and bottom rows) with pTDP-43-immunoreactive inclusions. pTDP-43-stained sections (Proteintech clone 22309-1-AP) and H&E-stained sections from Houston Methodist (lab of MDC) are shown in the left three panels for each patient. The right-most panel of each patient shows repeat pTDP-43 staining at the University of Kentucky using the protocol of that laboratory and a different pTDP-43 antibody (1D3 clone, 1:500). All images are photographed at 400x without magnification except for five images photographed at 200x (top row, two right-most panels; middle row, H&E section; bottom row, two left-most panels).

Online Resource Additional file 1

Figure S2. Multifocal, multi-segmental pTDP-43 inclusions in muscle of a c9ALS patient.

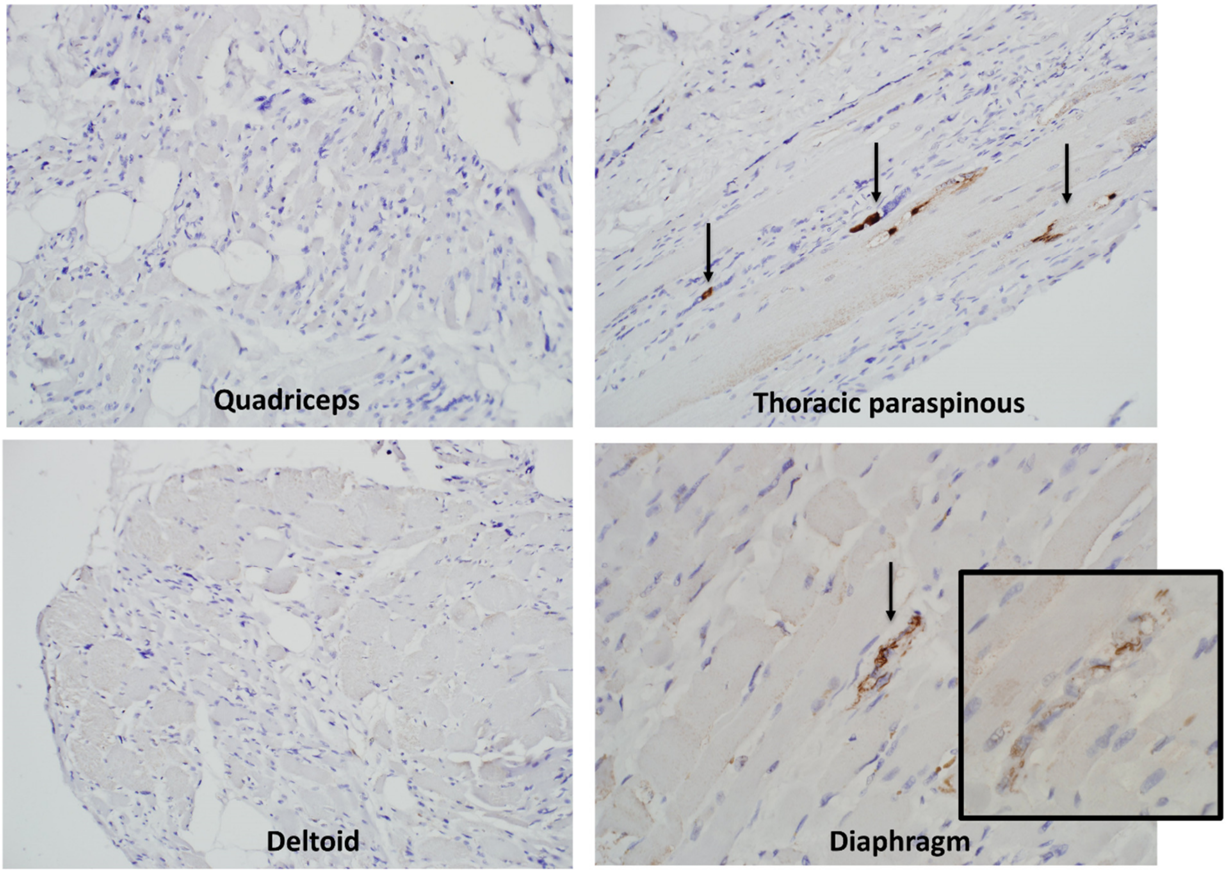

Figure S2 Four muscle samples from a recent autopsy of an ALS patient not included in this study. The patient had a clinical diagnosis of C9ALS and a positive family history of ALS (fALS). Examination of brain and spinal cord confirmed ALS and showed characteristic p62-immunoreactive and TDP-43-negative lesions in brain, consistent with C9ALS. Examination of seven muscle samples using p62 and pTDP-43 immunohistochemistry showed unequivocal pTDP-43-reactive lesions in muscle fibers of thoracic paraspinous and diaphragm muscle, an equivocal focus in sacral

paraspinous muscle (not shown), but no pTDP-43-positive lesions in cervical paraspinous muscle (not shown). The inset of diaphragm shows the same focus on a deeper histologic section, also stained for pTDP-43, further revealing the thread- and dash-like inclusions in this sample. All images are photographed are 200x except for diaphragm, which is photographed at 400x.

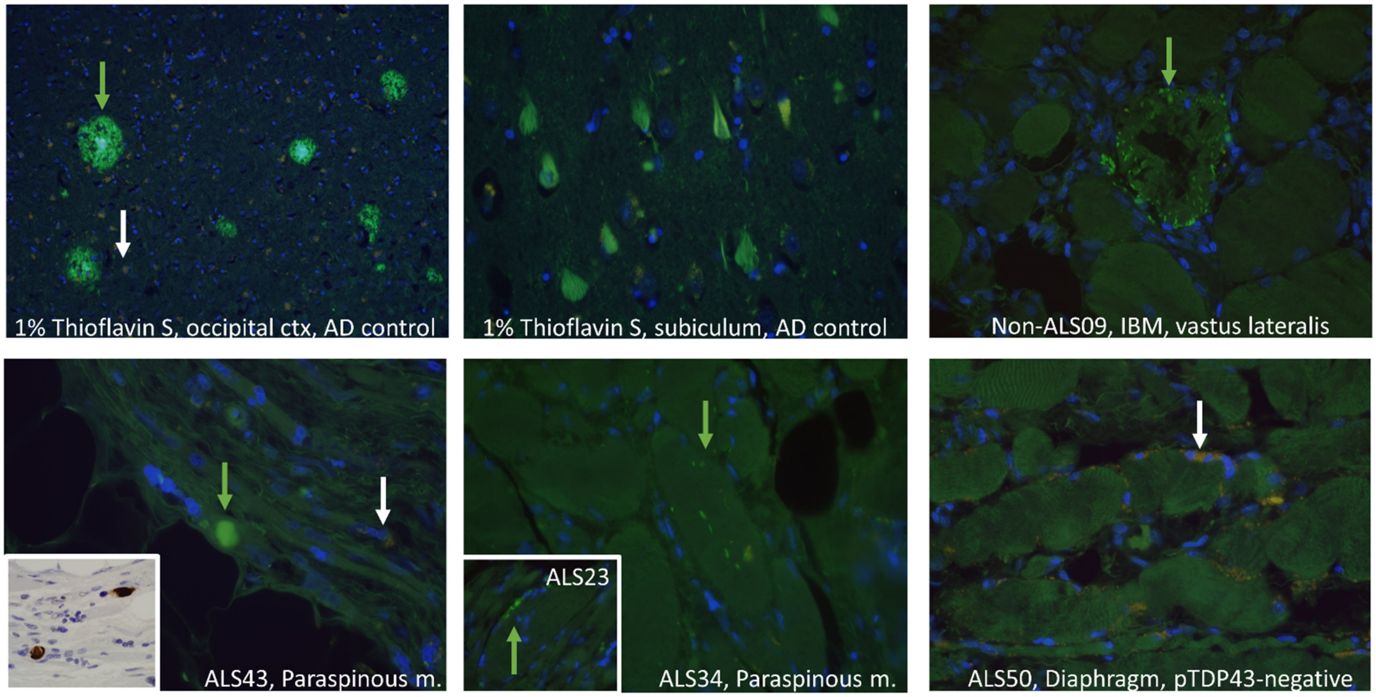

Figure S3 1% Thioflavin S staining was performed in Alzheimer's disease (AD) control tissue from occipital cortex and hippocampus/ subiculum (top left panel, 200x, and top middle panel, 400x), IBM muscle (top right, Non-ALS09, photographed at 600x), pTDP-43-positive ALS muscle samples with electron microscopy data shown in Fig 6 (ALS43, ALS34, ALS23), all photographed at 600x magnification, and a pTDP-43-negative ALS muscle with atrophy and abundant lipofuscin pigment (ALS50) (600x). Green arrows highlight thioflavin S-reactive material in all samples, whereas white

arrows indicate autofluorescent material, such as lipofuscin pigment (top left, bottom left, and bottom right panels), which appears yellow-orange in images combining FITC/ DAPI/ and TRITC filters (see Methods for detail on immunofluorescence microscopy). For ALS sample ALS43, the inset at lower left (600x) shows the similar shape of inclusions in this area by pTDP-43 immunohistochemistry.
